# Clinical phenotypes and short-term outcomes based on prehospital point-of-care testing and on-scene vital signs

**DOI:** 10.1038/s41746-024-01194-6

**Published:** 2024-07-24

**Authors:** Raúl López-Izquierdo, Carlos del Pozo Vegas, Ancor Sanz-García, Agustín Mayo Íscar, Miguel A. Castro Villamor, Eduardo Silva Alvarado, Santos Gracia Villar, Luis Alonso Dzul López, Silvia Aparicio Obregón, Rubén Calderon Iglesias, Joan B. Soriano, Francisco Martín-Rodríguez

**Affiliations:** 1 Faculty of Medicine. Universidad de Valladolid, Valladolid, Spain; 2 Emergency Department. Hospital Universitario Rio Hortega, Valladolid, Spain; 3 CIBER of Respiratory Diseases (CIBERES), Institute of Health Carlos III, Madrid, Spain; 4 Emergency Department. Hospital Clínico Universitario, Valladolid, Spain; 5 Faculty of Health Sciences, University of Castilla la Mancha, Talavera de la Reina, Spain; 6 Technological Innovation Applied to Health Research Group (ITAS Group), Faculty of Health Sciences, University of de Castilla-La Mancha, Talavera de la Reina, Spain; 7 Evaluación de Cuidados de Salud (ECUSAL), Instituto de Investigación Sanitaria de Castilla-La Mancha (IDISCAM), Talavera de la Reina, Spain; 8 Department of Statistics and Operative Research. Faculty of Medicine, University of Valladolid, Valladolid, Spain; 9 Universidad Europea del Atlántico, Santander, Spain; 10 Universidad Internacional Iberoamericana, Campeche, México; 11 Universidad de La Romana, La Romana, República Dominicana; 12 Universidad Internacional Iberoamericana Arecibo, Puerto Rico, USA; 13 Fundación Universitaria Internacional de Colombia, Bogotá, Colombia; 14 Universidade Internacional do Cuanza. Cuito, Bié, Angola; 15 Facultad de Medicina, Universidad Autónoma de Madrid, Madrid, Spain; 16 Servicio de Neumología; Hospital Universitario de La Princesa, Madrid, Spain; 17 Advanced Life Support, Emergency Medical Services (SACYL), Valladolid, Spain

**Keywords:** Outcomes research, Predictive markers

## Abstract

Emergency medical services (EMSs) face critical situations that require patient risk classification based on analytical and vital signs. We aimed to establish clustering-derived phenotypes based on prehospital analytical and vital signs that allow risk stratification. This was a prospective, multicenter, EMS-delivered, ambulance-based cohort study considering six advanced life support units, 38 basic life support units, and four tertiary hospitals in Spain. Adults with unselected acute diseases managed by the EMS and evacuated with discharge priority to emergency departments were considered between January 1, 2020, and June 30, 2023. Prehospital point-of-care testing and on-scene vital signs were used for the unsupervised machine learning method (clustering) to determine the phenotypes. Then phenotypes were compared with the primary outcome (cumulative mortality (all-cause) at 2, 7, and 30 days). A total of 7909 patients were included. The median (IQR) age was 64 (51–80) years, 41% were women, and 26% were living in rural areas. Three clusters were identified: *alpha* 16.2% (1281 patients), *beta* 28.8% (2279), and *gamma* 55% (4349). The mortality rates for *alpha*, *beta* and *gamma* at 2 days were 18.6%, 4.1%, and 0.8%, respectively; at 7 days, were 24.7%, 6.2%, and 1.7%; and at 30 days, were 33%, 10.2%, and 3.2%, respectively. Based on standard vital signs and blood test biomarkers in the prehospital scenario, three clusters were identified: *alpha* (high-risk), *beta* and *gamma* (medium- and low-risk, respectively). This permits the EMS system to quickly identify patients who are potentially compromised and to proactively implement the necessary interventions.

## Introduction

Emergency medical services (EMSs) must manage acute life-threatening illness as part of the standard workflow. EMS providers must perform timely decision-making without delay and in dynamic, critical scenarios. The quick targeting of high-risk patients represents a major challenge in prehospital care^1^, and new strategies to improve their timely recognition are being continuously implemented^2^. Accordingly, the application of scores, biomarkers, risk models, and other markers is becoming routine in clinical practice^3^.

In patients without a clear acute life-threatening illness, on-scene blood tests may assist in screening for hidden high-risk conditions, e.g., electrolyte disturbances, metabolic-endocrine diseases, respiratory failure, anemia, or renal insufficiency^4^. Point-of-care testing (POCT) allows blood test results, including venous or arterial blood gas levels, renal profile, glucose, lactate, hematocrit, hemoglobin, troponin, D-dimer, myoglobin, and international normalized ratio, to be obtained. The POCT provides EMS healthcare personnel a quick (few minutes) bedside analytical data, which was formerly reserved for hospital use exclusively, but now helps and supports the on-scene decision-making process^5^.

In addition, precision emergency medicine is a hot research area. In prehospital critical care, early warning scores, risk scales, and predictive models are commonly used to detect time-dependent diseases and their short- and long-term prognoses^6,7^. Likewise, phenotypes are increasingly used to identify certain pathophysiological conditions in hospitals^8,9^, and they are applied in the prehospital setting^10,11^.

To our knowledge, there is limited evidence on phenotypes in prehospital care^12,13^. Accordingly, we aimed to develop clustering-derived phenotypes in patients with acute life-threatening illnesses based on vital signs and biomarkers collected by EMS upon initial emergency care. Furthermore, we aimed to determine the short- and midterm prognoses of these patients and the diseases associated with each phenotype.

## Results

**Fig. 1:**
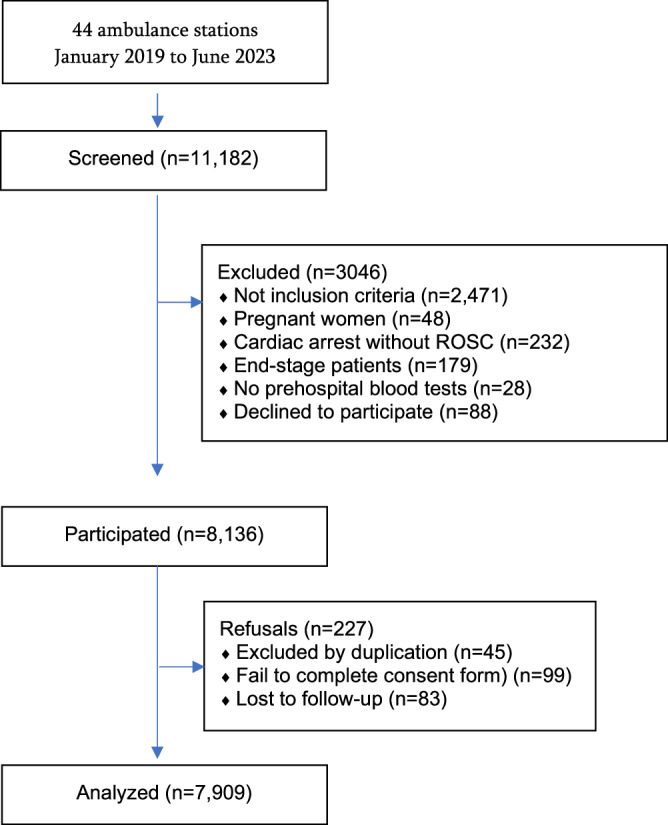
Study flowchart. ROSC recovery of spontaneous circulation.

From an original population of 11,182 patients, 8136 were considered eligible, and 7909 (97.2%) subjects satisfied the inclusion criteria and were included in the final cohort analysis (Fig. 1). The median (IQR) age was 64 (51–80) years, 41% were women, and 26% were living in rural areas (Table 1). Clinical characterization via unsupervised machine learning revealed three clinical phenotypes that exhibited marked differences.

**Table 1:** Clinical and biomarker baseline patient characteristics

		Phenotype			
	Total	Alpha	Beta	Gamma	*p* value^b^
No. (%) with data^a^	7909 (100)	1281 (16.2)	2279 (28.8)	4349 (55)	N.A.
*Sociodemographic variables*					
Sex at birth, female	3280 (41)	534 (41.7)	906 (39.8)	1840 (42.3)	0.132
Age, year	64 (51–80)	74 (62–84)	72 (56–82)	62 (47–78)	<0.001
Age groups, year					
18–49	1679 (21.2)	148 (11.6)	371 (16.3)	1253 (28.8)	<0.001
50–74	3128 (39.5)	501 (39.1)	887 (38.9)	1740 (40)	
>75	3009 (38.0)	632 (49.3)	1021 (44.8)	1356 (31.2)	
Zone, rural	2053 (26.0)	380 (29.7)	564 (24.7)	1109 (25.5)	0.031
Transfer, ALS	2846 (36.0)	978 (76.3)	739 (32.4)	1804 (41.5)	<0.001
Nursing homes	808 (10.2)	278 (21.7)	269 (11.8)	261 (6)	<0.001
*On-scene vital signs*					
Respiratory rate, breaths/min	18 (14–23)	26 (17–33)	18 (15–24)	17 (14–19)	<0.001
Oxygen saturation, %	96 (94–98)	86 (76–94)	95 (92–97)	98 (96–99)	<0.001
Fraction of inspired oxygen, %	0.21 (0.21–0.21)	0.21 (0.21–0.31)	0.21 (0.21–0.21)	0.21 (0.21–0.21)	<0.001
SaFi	459 (442–466)	343 (257–398)	452 (438–462)	467 (457–471)	<0.001
Systolic blood pressure, mmHg	134 (114–153)	129 (99–155)	133 (112–152)	136 (119–153)	<0.001
Diastolic blood pressure, mmHg	79 (65–90)	72 (55–89)	77 (63–90)	80 (69–91)	<0.001
Mean blood pressure, mmHg	96 (93–110)	92 (71–110)	96 (81–110)	99 (87–111)	<0.001
Heart rate, beats/min	85 (70–104)	103 (80–120)	97 (77–121)	79 (67–90)	<0.001
Temperature, °C	36.1 (35.9–36.6)	36.2 (35.8–36.8)	36.1 (35.9–36.7)	36 (35.9–36.5)	<0.001
Glasgow coma scale, points					
Ocular	4 (4–4)	4 (2–4)	4 (4–4)	4 (4–4)	<0.001
Verbal	5 (5–5)	5 (2–5)	5 (5–5)	5 (5–5)	<0.001
Motor	6 (6–6)	6 (4–6)	6 (6–6)	6 (6–6)	<0.001
MEWS categories					<0.001
low risk (0–1)	4297 (54.3)	247 (19.3)	863 (37.9)	3187 (73.3)	
intermediate risk (2–3)	1967 (24.8)	327 (25.5)	42 (32.6)	898 (20.6)	
high risk (≥4)	1645 (20.7)	707 (55.2)	674 (29.6)	264 (6.07)	
ISS^c^	2 (1–9)	16 (9–16)	4 (1–16)	1 (1–4)	<0.001
*Prehospital blood analysis*					
pH	7.38 (7.33–7.42)	7.31 (7.14–7.38)	7.37 (7.32–7.42)	7.39 (7.36–7.42)	<0.001
pCO2, mmHg	40 (34–46)	48 (38–67)	40 (34–46)	39 (33–44)	<0.001
pO2, mmHg	31 (23–40)	23 (17–35)	30 (22–41)	32 (23–41)	<0.001
Bicarbonate, mEq	23 (21–26)	22 (18–27)	23 (20–26)	24 (22–27)	<0.001
Base excess (ecf), mmol/L	0.4 (−2.4; 1.9)	−1.8 (−7.2; 2.4)	−0.3 (−3; 1.7)	0.7 (−1.6; 1.9)	<0.001
TCO2, mmol/L	26 (23–29)	27 (22–34)	26 (22–29)	26 (23–28)	<0.001
Sodium, mmol/L	139 (137–141)	139 (136–141)	139 (136–141)	139 (137–141)	<0.001
Potassium, mmol/L	4.1 (3.8–4.5)	4.2 (3.9–5)	4.1 (3.8–4.6)	4.1 (3.8–4.4)	<0.001
Calcium, mmol/L	1.14 (1.08–1.21)	1.14 (1.04–1.22)	1.14 (1.08–1.21)	1.14 (1.08–1.21)	0.039
Chlorine, mmol/L	103 (100–106)	103 (100–107)	103 (100–106)	103 (100–105)	0.011
Hematocrit, %	42 (39–45)	41 (36–45)	41 (38–45)	42 (39–45)	<0.001
Hemoglobin, g/dL	14.1 (12.8–15.7)	13.8 (12.1–15.7)	14 (12.6–15.7)	14.2 (13–15.7)	<0.001
Glucose, mg/dL	126 (104–160)	183 (130–275)	160 (120–197)	113 (99–132)	<0.001
Lactate, mmol/L	2.14 (1.28–3.29)	3.29 (2.13–6.29)	2.43 (1.59–3.74)	1.83 (1.13–2.82)	<0.001
Creatinine, mg/dL	0.91 (0.76–1.21)	1.21 (0.88–1.86)	0.96 (0.79–1.28)	0.86 (0.75–1.08)	<0.001
Blood urea nitrogen, mg/dL	16 (12–23)	24 (16–37)	18 (13–26)	14 (11–20)	<0.001
Osmolarity, mOsm/kg	291 (287–297)	298 (290–307)	294 (288–299)	290 (286–294)	<0.001
GAP anion, mmol/L	11.6 (8.3–15.2)	11.8 (7.5–16.6)	11.8 (8.3–15.6)	11.5 (8.4–14.6)	0.026
Urinary anion, mmol/L	40.1 (37.1–42.9)	39.9 (36.1–43.2)	40 (36.8–42.8)	40.3 (37.5–42.8)	0.002
Potassium anion, mmol/L	15.8 (12.5–19.3)	16.2 (11.9–21.3)	16 (12.6–17.9)	15.7 (12.6–18.7)	<0.001

*NA* not applicable, *ALS* advanced life support, *SaFi ratio* pulse oximetry saturation/fraction of inspired oxygen ratio, *pCO*_*2*_ partial pressure of carbon dioxide, *pO2* partial pressure of oxygen, *TCO*_*2*_ total carbon dioxide content, *ISS* Injury Severity Score, *MEWS* Modified Early Warning Score.

^a^Values are expressed as the total number (percentage) and median (25th percentile-75th percentile), as appropriate.

^b^The Mann‒Whitney *U* test or chi-squared test was used as appropriate.

^c^Note that ISS was only determined in trauma patients.

The clustering procedure was preceded by a reduction in dimensionality. As shown in Supplementary Fig. 1, the first three dimensions explained 81.9% of the variance. The principal component analysis output was subsequently used for the clustering procedure, as shown in Supplementary Fig. 2. The most parsimonious clustering model was the ellipsoidal, varying volume, shape, and orientation (VVV) model. Moreover, when the number of clusters was increased, the clustering model was stable, and no major difference in the BIC was found, suggesting that the VVV model and the clinically selected number of clusters were supported by the BIC results. The cluster results and an explanation of clinical criteria for the selection of the number of clusters are shown in Supplementary Fig. 3.

*The alpha* phenotype was found in 16.2% (1281) of the patients, with a median age of 74 years, 41.7% (534 patients) female sex, an ALS evacuation rate of 76.3% (978 patients) and a nursing home origin of 21.7% (278 patients). The *beta* phenotype accounted for 28.8% (2279) of the patients, with a median age of 72 years and 39.8% (906 patients) female sex; 32.4% of the ALS patients evacuated and 11.8% (269 patients) evacuated from the origin of the nursing home. Overall, the *gamma* phenotype represented 55% (4349) of the patients, with a median age of 62 years; 42.3% (1840) of whom were females; 41.5% (1804 patients) who underwent ALS transfer; and 6% (261 patients) who were from nursing homes (Table 1).

On-scene vital signs also showed significant differences between the clusters. The *alpha* phenotype exhibited increased respiratory and cardiac rates and decreased saturation, SaFi, blood pressure and Glasgow coma scale (*p* < 0.001 in all patients). Differences across phenotypes were also evident in blood biomarkers, with significant differences among other parameters in pH, partial pressure of carbon dioxide, lactate, creatinine, and glucose (*p* < 0.001 in all) (Table 1).

**Fig. 2:**
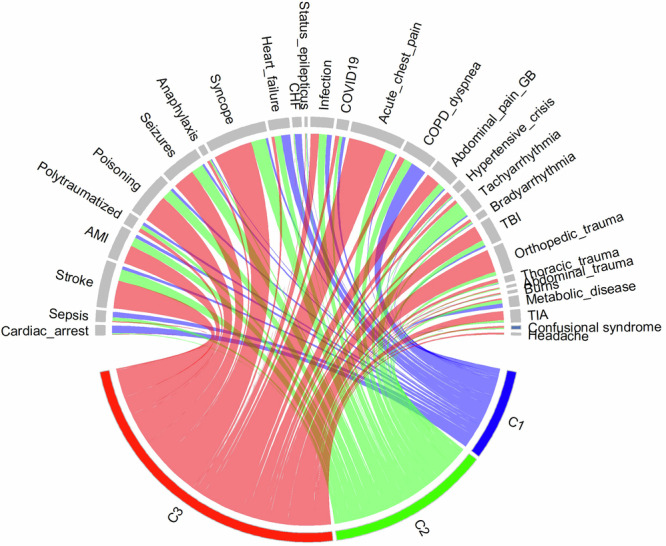
Distribution of suspected prehospital diagnoses. Chord diagram representing the distribution of suspected prehospital diagnoses in each cluster. The blue line = alpha (C1), the green line = beta (C2), and the red line = gamma (c3).

The distribution of suspected prehospital diagnoses in each cluster is shown in Fig. 2. Patients with acute life-threatening diseases were assigned by the unsupervised clustering method to the *alpha* phenotype and a priori less severe diseases or nonspecific syndromic conditions to the other two clusters. Accordingly, patients with the *alpha* phenotype were characterized by cardiac arrest, heart failure (including congestive heart failure) and dyspnea, followed by febrile syndrome, sepsis, and COVID-19; those with the *beta* phenotype displayed several heterogeneous conditions: tachyarrhythmias, syncope, seizures, stroke, acute chest pain and poisoning; and those with the *gamma* phenotype presented syncope, acute chest pain, stroke, poisoning, orthopedic trauma, and seizures.

**Fig. 3:**
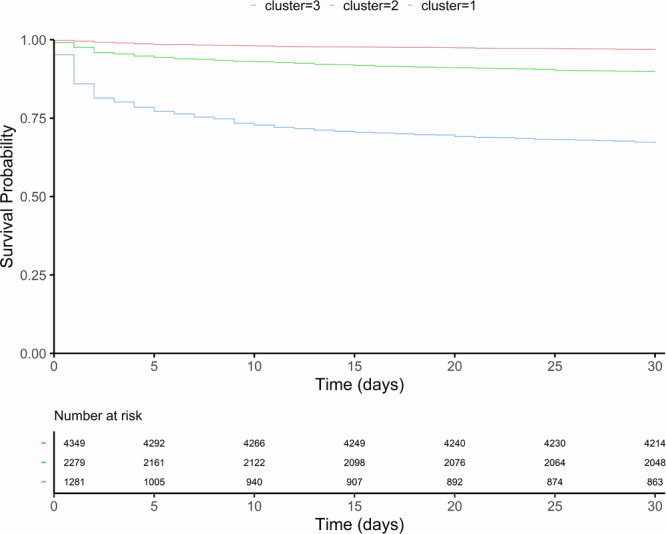
Phenotype survival. Survival curve of each phenotype. The blue line = alpha, the green line = beta, and the red line = gamma.

The 2-day mortality rates were 18.6%, 4.1%, and 0.8% for the *alpha*, *beta* and *gamma* phenotypes, respectively. Moreover, 24.7%, 6.2%, and 1.7% of the patients died within 7 days, and 33%, 10.2%, and 3.2% died within 30 days (Table 2). In addition to mortality disparities, *the alpha* phenotype stood out due to an increased requirement for on-scene advanced life support interventions, associated burden of comorbidities, and major ICU admissions. Survival analysis revealed that the hazard ratios (HRs) for mortality in patients with the *beta* and *alpha* phenotypes were 3.37 (95% CI: 2.73–4.16) and 12.8 (95% CI: 10.6–15.6), respectively, when *gamma* was used as a reference (Supplementary Table 1). As shown in Fig. 3, the highest mortality in the *alpha* phenotype occurred immediately, while the *beta* and *gamma* phenotypes separated within the first five days. All three curves slowed (shallow slopes) as time progressed. Supplementary Fig. 4 shows the survival curves of the three clustering-derived phenotypes as compared to low, medium, and high-risk categories of modified early warning score (MEWS). The mortality curves of each phenotype matched the mortality curve of each risk category of MEWS, this is, *gamma* phenotype was parallel to low-risk, *beta* to intermediate risk, and *alpha* to high risk, but always with phenotypes curves below the MEWS ones.

**Table 2:** Principal outcomes and other determinants

	Phenotype			
	Alpha	Beta	Gamma	*p* value^b^
No. (%) with data^a^	1281 (16.2)	2279 (28.8)	4349 (55)	N.A.
*Support on-scene*				
NIMV	246 (19.2)	41 (1.8)	6 (0.1)	<0.001
IMV	260 (20.3)	130 (5.7)	95 (2.2)	<0.001
Pacemaker	26 (2)	34 (1.5)	42 (1)	0.007
Cardioversion	19 (1.5)	60 (2.6)	3 (0.1)	<0.001
Defibrillation	75 (5.9)	12 (0.5)	8 (0.2)	
Intravenous medication, quantity				
No medication	52 (4.1)	416 (18.3)	1316 (30.3)	<0.001
1	122 (9.5)	587 (25.8)	1258 (28.9)	
2	176 (13.7)	427 (18.7)	837 (19.2)	
3	204 (15.9)	363 (15.9)	467 (10.7)	
4	219 (17.1)	243 (10.7)	272 (6.3)	
5	222 (17.3)	133 (5.8)	125 (2.9)	
6	163 (12.7)	66 (2.9)	57 (1.3)	
7 or more	123 (9.6)	44 (1.9)	17 (0.4)	
Vasoactive agents	145 (11.3)	36 (1.6)	11 (0.3)	<0.001
*Suspected prehospital diagnoses*				
Abdominal pain/GB	22 (1.7)	107 (4.7)	213 (4.9)	<0.001
Abdominal trauma	2 (0.2)	13 (0.6)	16 (0.4)	
Acute chest pain	19 (1.5)	158 (6.9)	578 (13.3)	
Acute myocardial infarction	52 (4.1)	134 (5.9)	278 (6.4)	
Anaphylaxis	16 (1.2)	24 (1.1)	41 (0.9)	
Bradyarrhythmia	9 (0.7)	31 (1.4)	44 (1)	
Burns	6 (0.5)	9 (0.4)	19 (0.4)	
Cardiac arrest	117 (9.1)	14 (0.6)	7 (0.2)	
Confusional syndrome	3 (0.2)	18 (0.8)	28 (0.6)	
Congestive heart failure	104 (8.1)	15 (0.7)	2 (0)	
COPD/dyspnea	237 (18.5)	125 (5.5)	86 (2)	
Headache	2 (0.2)	1 (0)	32 (0.7)	
Heart failure	137 (10.7)	102 (4.5)	46 (1.1)	
Hypertensive crisis	10 (0.8)	18 (0.8)	87 (2)	
Infection/febrile syndrome	74 (5.8)	120 (5.3)	125 (2.9)	
Metabolic disease	64 (5)	56 (2.5)	22 (0.5)	
Orthopedic trauma	3 (0.2)	53 (2.3)	346 (8)	
Poisoning	41 (3.2)	155 (6.8)	419 (9.6)	
Polytraumatized	44 (3.4)	58 (2.5)	60 (1.4)	
SARS-CoV-2	50 (3.9)	49 (2.2)	60 (1.4)	
Seizures	35 (2.7)	182 (8)	329 (7.6)	
Sepsis	82 (6.4)	56 (2.5)	17 (0.4)	
Status epilepticus	8 (0.6)	15 (0.7)	9 (0.2)	
Stroke	49 (3.8)	175 (7.7)	420 (9.7)	
Syncope	35 (2.7)	224 (9.8)	589 (13.5)	
Tachyarrhythmia	23 (1.8)	234 (10.3)	50 (1.1)	
Thoracic trauma	7 (0.5)	21 (0.9)	55 (1.3)	
Transient ischemic attack	3 (0.2)	32 (1.4)	137 (3.2)	
Trauma brain injury	31 (2.4)	79 (3.5)	234 (5.4)	
*Hospital outcomes*				
aCCI, points	6 (4–9)	5 (3–7)	3 (1–5)	<0.001
Inpatient	1108 (86.5)	1345 (59)	1793 (41.2)	<0.001
ICU-admission	339 (26.5)	281 (12.3)	273 (6.3)	<0.001
ACCU-admission	124 (9.7)	200 (8.8)	325 (7.5)	0.021
Stroke unit-admission	15 (1.2)	110 (4.8)	289 (6.6)	<0.001
Mortality				
2-day	238 (18.6)	93 (4.1)	33 (0.8)	<0.001
7-day	316 (24.7)	142 (6.2)	74 (1.7)	<0.001
30-day	423 (33)	233 (10.2)	137 (3.2)	<0.001

*NA* not applicable, *NIMV* noninvasive mechanical ventilation, *IMV* invasive mechanical ventilation, *GB* gastrointestinal bleeding, *COPD* chronic obstructive pulmonary disease, *SARS-CoV-2* severe acute respiratory syndrome coronavirus 2, *aCCI* age-adjusted Charlson comorbidity index, *ICU* intensive care unit, *ACCU* acute cardiac care unit.

^a^Values are expressed as the total number (percentage) and median (25th percentile-75th percentile), as appropriate.

^b^The Mann‒Whitney U test or chi-squared test was used as appropriate.

Finally, a clustering of the *gamma* phenotype was performed (Supplementary Fig. 5, 6, 7). The three *gamma* subclusters (*n* = 1321, 1704 and 1324 for *gamma* #1, #2, and #3, respectively) showed that mortality was higher for *gamma* #1 (1.14%, 2.8%, and 5.37%, at 2, 7 and 30-day mortality), followed by *gamma* #3 (1.06%, 2.19%, and 3.39%, at 2, 7 and 30-day mortality), the *gamma* #2 phenotype presented the lowest mortality rate (0.23%, 0.47%, and 0.82%, at 2, 7 and 30-day mortality) (Supplementary Table 2).

## Discussion

Our study described different phenotypes with increasing severity based only on on-scene variables and biomarkers in adults with unselected acute diseases managed by EMS who were evacuated with priority to the ED. The alpha and beta phenotypes identified those patients at risk of clinical worsening in a more appropriate way than the intermediate and high-risk of MEWS, making these clusters more valuable for triage. This study paves the way for applying standardized prehospital laboratory tests and routine vital signs to determine bedside phenotypes. Phenotyping to target critical care and support the decision-making process might become commonplace in clinical practice. This methodology is already well established for sepsis, chronic obstructive pulmonary disease/asthma, and heart failure^9,14^. More recently, it has been used to develop real-time solutions against COVID-19^15^. Nevertheless, phenotyping during prehospital critical care is emerging tentatively^12,16,17^.

Based on 30 variables (sociodemographic, clinical, and analytical biomarkers) collected during prehospital care and blinded to the main dependent outcome, three clustering-derived phenotypes were identified. The *alpha* phenotype was characterized by a compromised clinical condition (tachypnea, desaturation, impaired SaFi, lower blood pressure, tachycardia, and a poorer consciousness level), associated with acidosis, hypercapnia, negative base excess, hyperlactacidemia, an abnormal renal profile (creatinine and blood urea nitrogen rises) and hyperglycemia; such patients presented a marked dependence over time for on-scene life support interventions, the greatest rates of ICU admissions, and mortality (3-times greater for the *beta* phenotype and 10-times greater for the *alpha* phenotype, both compared to *gamma*). Next, patients in the *beta* phenotype were characterized by an improved acid‒base balance, increased blood oxygen, mild hyperlactacidemia, a renal profile that returned to target ranges, and mild hyperglycemia. Finally, most of the *gamma* phenotype patients presented results within normal ranges.

As previously mentioned, the suspected prehospital diagnoses vary largely by phenotype; *the alpha* phenotype is characterized by severe heart disease and other conditions associated with high short- and long-term morbidity and mortality^18^. The *beta* phenotype conditions were highly heterogeneous. Finally, the *gamma* phenotype included a priori less severe diseases or nonspecific syndromic conditions. Our results aligned well with previous evidence, pooling in one cluster of critically ill patients^14,19^.

Consistently, *the alpha* phenotype was associated with high-level on-scene advanced life support interventions, ICU admissions, frequent advanced airway management and intravenous medication. This finding contrasted with the findings of *beta phenotypes*, particularly with the *gamma* phenotype, which requires less use of health services, which even more critical for the gamma subcluster #2, presenting a very low mortality rate of less than 1%, therefore, requiring lower attention by the EMS. Clinical evidence suggests an association between unplanned mechanical ventilation and mortality, just as concomitant administration of medication correlates with a worse prognosis^20^, thus suggesting that the group of patients who meet these criteria are in the most critical phenotype category. As expected, the cluster with the poorest outcomes (*alpha*) mostly included elderly patients and was more burdened by comorbidities. Several risk scores consider age and comorbidities as vulnerability indicators, such as the aCCI^21^. Frailty syndrome is a well-described multidimensional condition that, despite individual variability, constitutes a focal point directly related to poor outcomes. As age and comorbidities progress, physiological and psychosocial reserves may be jeopardized, enhancing clinical vulnerability^22^.

An innovative objective of this study was to conduct phenotyping with ultra-early (first contact with patients by the EMS staff) analytical data based on prospective and standardized POCT. From primitive capillary glucometers to current POCTs, technological advances have favored the production of novel devices available on-scene with reduced dimensions that are portable, robust and highly reliable, making them an ideal solution for deployment in ambulances^3^. Due to the support provided by POCT, EMS providers obtain crucial medical data quickly during the turnaround period; otherwise, the data are retrieved only from the hospital. We demonstrated that objective and structured clinical evaluation combined with biomarker testing in acute life-threatening diseases can guide targeted life support interventions on-scene or en route and optimize decision-making processes in prehospital critical care, all of which are aligned with international guideline recommendations^5,23^.

Phenotyping has begun to be incorporated in particular diseases, mainly in the hospital setting. García-Vidal, C. et al.^24^ developed a system for the timely detection of high-risk patients during the first wave of the last COVID-19 pandemic. Using artificial intelligence techniques, they were able to identify three phenotypes: inflammation, superinfection and thrombotic events. Their system analyzed data in real time, allowing early decisions and quick personalized treatments, with a 90% prediction of patient evolution and a 50% reduction in mortality. Komorowski, M. et al.^25^ developed the “AI Clinician”, a computational model based on reinforcement learning capable of dynamically suggesting optimal treatments for ICU patients with sepsis. Their model uses variables very similar to those proposed in our model. The AI Clinician was able to suggest individualized and clinically interpretable treatment strategies for sepsis. In an independent cohort, patients who received the treatments suggested by the AI Clinician had the lowest mortality rate. In the prehospital setting, Kang, D. et al.^26^, using deep learning algorithms, predicted the need for critical care by the EMS, with an AUROC of 0.867, outperforming conventional triage tools and early warning scores.

Unfortunately, prehospital care studies, such as the one from Kang et al., are exceptions, in part, due to the complexity of out-of-hospital work, hindering the implementation of EMS systems. The on-scene workflow, rushed decision-making, and ongoing dramatic interventions make inferring the patient’s phenotype impossible for EMS providers without support. One possible way to bridge this gap is to implement the algorithm developed to derive phenotypes in EMS electronic medical records. In this way, in real time and at the bedside, the EMS provider could have access to the information, supporting the decision-making process. This a priori difficult adoption of phenotyping could follow the example of scores, which are routinely employed in health services, e.g., body mass index, Glasgow coma scale, CHA2DS2-VASc score for atrial fibrillation stroke risk, etc. EMSs are not an exception since the use of early warning scores is a reality and mandatory for the decision-making process. Therefore, since EMS professionals are accustomed to work with scores, the implementation of phenotyping systems in the EMS could be a straightforward process.

The main strength of this study is that, by means of a free-scale machine learning methodology, we identified a phenotype, *alpha*, which comprises medically challenging conditions, with some degree of frailty and evident clinical disorders (impaired respiratory capacity, hemodynamic unsteadiness, neurological deterioration, lactic acidosis, hyperglycemia, etc.). Sixteen percent of patients, those typically requiring several advanced life support interventions on-scene, with a large proportion of inpatients admitted to the ICU, were ultimately associated with elevated mortality. Additionally, this method allowed to characterize patients which are not easy to identify such as those from beta, gamma, and even gamma subclusters, increasing the capability of the EMS to identify true negative patients. This zero-minute flagging of high-risk patients, based not only on standard vital signs but also on optimal support from blood test biomarkers, empowers the EMS system to recognize patients potentially compromised and to proactively implement the necessary interventions^27^. In this sense, artificial intelligence represented a breakthrough, emerging phenotyping as a flexible and useful solution with a proven risk-based case matching capability, allowing massive data analysis to classify high-risk patients as sentinel events^26^. Other strengths of our study include its large size, population size based on few exclusion criteria, and real-world setting.

### Limitations of the study

However, a number of limitations are worth considering. First, a convenience sample was compiled. To minimize bias, a dual strategy was employed. All adult patients were screened for eligibility on a 24/7/365 basis; in addition, patients with various ALS types from urban and rural locations and from hospitals with diverse capabilities (one minor general district hospital and three university tertiary hospitals) were included in the study. Second, the data extractors were unblinded. To prevent crossover connections, the EMS providers had no access to the hospital follow-up data; vice versa, the hospital investigators were unaware of the prehospital care data; only the principal investigator and the data manager received full access to the master database and the phenotyping output. Third, the EMS medical records are still paper-based and not yet electronic. Manual review of the patient medical records attended by the EMS (the current reference standard for identifying patient cohorts) demands a significant amount of time and resources. Considerable efforts are being made by the Public Health system to implement a prehospital electronic health record system involving both the BLS and ALS, with operational capacity for real-time transmission of all the information to the ED. Fourth, despite the rapid expansion of POCT in numerous EMS systems around us, this technology has not been regularly implemented in all ambulances or all ALS wards. Finally, the study was carried out before and concurrently with the ongoing COVID-19 pandemic. At the peak of the first wave of the pandemic, EMS activation for acute life-threatening diseases declined drastically, and the extent of the effect of COVID-19 on the physiological and psychosocial reserve of surviving patients is unclear. More research is needed to determine the excess mortality due to non-COVID-19 pathology in the prevaccination stage, and in addition, the role of COVID-19 in increasing clinical vulnerability in the medium and long term should be explored.

In summary, based on data collected exclusively in prehospital care, unselected acute disease patients managed by EMS and transferred to the ED can be categorized into three phenotypes with different clinical and prognostic implications. At the first point of care, EMS staff can identify the risk level, avoid underrated hidden unresolved health conditions and characterize complex or atypical clinical presentations. Identifying patients with an *alpha* phenotype from the initial moments of assistance allows the development of a personalized strategy, tailoring the level of support and resources to individual situations, or even determining the most appropriate course of action for each patient. This knowledge provides valuable information for bedside decision-making from the outset to design the best possible care strategy tailored to the individual case.

## Methods

### Study design and setting

A prospective, multicenter, EMS-delivered, ambulance-based cohort study was conducted with adults with unselected acute diseases (assistance by an advanced life support unit -ALS-) managed by EMS who were evacuated with priority discharge to the ED from January 1, 2020, to June 30, 2023.

The study involved the use of a 1–1–2 emergency coordination center, six ALS units, 38 basic life support (BLS) units, and four hospitals in Salamanca, Segovia and Valladolid (Spain), comprising a population of 995,137 inhabitants and comprising urban and rural communities. The public health system managed and coordinated all the facilities. BLSs include two emergency medical technicians (EMTs); ALSs are made by an emergency registered nurse (ERN) and a physician, operating all EMS providers in compliance with life support guidelines.

Patients were prospectively included uninterruptedly from two studies conducted under identical research protocols, the “HITS study” (ISRCTN48326533) and the preBIO study” (ISRCTN49321933), which were approved by the institutional review board of the Public Health Service and followed the STrengthening the Reporting of OBservational studies in Epidemiology (STROBE) statement (supplementary material Note 1)^28^. Informed consent was obtained from all human participants.

### Population

Adults (>18 years) with unselected acute illnesses were screened for eligibility consecutively 24/7/365 by the EMS. Additionally, following an evaluation by an ALS physician, to be included in the study, patients had to be mandatorily referred to the emergency department (ED), either at the BLS or at the ALS.

Minors, pregnant women (evident or probable), cardiac arrest without recovery of spontaneous circulation on-scene, end-stage patients (documented by a report), impossibility to obtain prehospital blood tests (e.g., difficulty to establish venous access, breakdown of blood testing device), and no informed consent were excluded. Patients requiring prehospital care and already registered in the database for previous care were excluded.

### Outcome

The principal outcome was cumulative mortality (all-cause) at 2, 7, and 30 days. The secondary variables considered included on-scene life support interventions (advanced airway management, defibrillation or pacemaker application, and intravenous medication delivery), suspected prehospital diagnoses (29 different subcategories), hospital outcomes (inpatient, intensive care unit admission), and 17 comorbidities needed to calculate the age-adjusted Charlson comorbidity index (aCCI).

### Data collection and processing

The EMS providers received prior face-to-face training on the implementation of the research protocol and standardized data entry into the database.

Covariates included sociodemographic variables (sex at birth and age); on-scene vital signs (respiratory rate, oxygen saturation, blood pressure, heart rate, temperature, and Glasgow coma scale); and prehospital blood analysis (pH, bicarbonate, excess bases, sodium, potassium, chloride, calcium, hemoglobin, hematocrit, creatinine, blood urea nitrogen, glucose, lactate, osmolarity, GAP anion, urinary anion, and potassium anion), which were obtained by the ERN. Measurements were collected immediately upon starting prehospital care on the first patient encounter. Vital signs were obtained via a LifePAK® 15 monitor-defibrillator (Physio-Control, Inc., Redmond, USA), and blood tests were performed by means of an Epoc® analyzer (Siemens Healthcare GmbH, Erlangen, Germany). The respiratory rate was monitored by direct observation and counting of breathing cycles for half a minute; in the case of very shallow or difficult breathing, the respiratory rate was measured by direct auscultation. Oxygen therapy (by any method) was also administered at the time of the patient’s diagnosis of ALS; once the fraction of inspired oxygen was known, the pulse oximetry saturation/fraction of inspired oxygen ratio (SaFi) was calculated.

After a 30-day follow-up period, data on mortality, comorbidities and hospital admissions were collected by reviewing the patients’ electronic medical records. The data were recorded electronically in a database specifically designed for this purpose, recording the prehospital care variables. Access was provided by individual passwords and double authentication. After the data were cleaned (logic, range and consistency tests), a total of 54 variables were analyzed. Once the data were linked, patient identifiers were anonymized.

### Statistical analysis

Descriptive and bivariate statistics for the outcome variables were assessed by the *t* test, the Mann‒Whitney *U* test or the chi-square test, whenever appropriate. Absolute values and percentages were used for categorical variables, and median interquartile ranges (IQRs) were used for continuous variables that were not normally distributed. The clustering procedure was as follows: First, a reduction in dimensionality (principal component analysis) was used to reduce the number of variables. The most parsimonious clustering model was selected by the Bayesian information criterion (BIC) to perform Gaussian mixture modeling for model-based clustering. Since the clusters were obtained from the same unsupervised method, all resulted from the same set of variables. The number of clusters was fixed to three based on clinical criteria. Finally, each cluster was explored by including the outcomes, life support interventions, suspected prehospital diagnoses, and aCCI. Finally, a survival analysis was performed according to each phenotype; this was the univariate comparison between each independent variable and the outcome, assessed by the log-rank test, and the survival curve according to clusters was obtained using the Kaplan‒Meier method (KM).

The data were collected and registered in a database generated with the IBM SPSS Statistics for Apple version 20.0 software. (IBM Corp, Armonk, NY, USA). The caseload entry system was tested to delete unclear or ambiguous values and to verify the adequacy of the data gathering system. Missing values were random; therefore, a listwise deletion method was used since it does not induce biased means, variances or regression weight modifications. The sample size needed for the clustering studies has been recently estimated^29^. Due to the characteristics of the clustering procedure, the phenotypes derived from clustering are driven by large effect sizes or by the accumulation of small effect sizes among the multiple variables analyzed, and there is no effect of the covariance structure difference. Therefore, a small sample size (e.g., *N* = 20), as stated in ref. ^29^, allows large cluster separations.

All calculations and analyses were performed by our own codes, R packages (mclust^30^) and base functions in R, version 4.2.2 (http://www.R-project.org; the R Foundation for Statistical Computing, Vienna, Austria).

### Inclusion and ethics statement

All collaborators of this study have fulfilled the criteria for authorship required by Nature Portfolio journals have been included as authors, as their participation was essential for the design and implementation of the study. Roles and responsibilities were agreed among collaborators ahead of the research. This work includes findings that are locally relevant, which have been determined in collaboration with local partners. This research was not severely restricted or prohibited in the setting of the researchers, and does not result in stigmatization, incrimination, discrimination, or personal risk to participants. Local and regional research relevant to our study was taken into account in citations.

### Reporting summary

Further information on research design is available in the Nature Research Reporting Summary linked to this article.

### Supplementary information

Supplementary Information

Reporting Summary

## Data Availability

The datasets used and/or analyzed during the current study are available from the corresponding author upon reasonable request.

## Code availability

The underlying code for this study [and training/validation datasets] is not publicly available but may be made available to qualified researchers upon reasonable request from the corresponding author.
