# Supplementary material for: Clinical phenotypes and short-term outcomes based on prehospital point-of-care testing and on-scene vital signs
Source: NPJ Digit Med. 2024 Jul 24;7:197. doi: 10.1038/s41746-024-01194-6 (PMC11269726; doi:10.1038/s41746-024-01194-6)
Supplement: Supplementary file 1 — Supplementary Information [file 41746_2024_1194_MOESM1_ESM.pdf]

Supplementary data

Table of contents

Supplementary methods……………………………………………………………3
- Supplementary Note 1: Strengthening the Reporting of Observational Studies in Epidemiology (STROBE) Statement.……..……………………………….…3

Supplementary Results…………………………………………………………..5
- Supplementary Figure 1: Percentage of explained variances by the dimensions……………………………………………………………….5
- Supplementary Figure 2: Bayesian Information Criterium for the different clustering number and methods…………………………………………………6
- Supplementary Figure 3: Clustering-derived phenotypes……………………...7
- Supplementary Table 1: Univariate analysis of mortality……………………..8
- Supplementary Figure 4: Survival curves of phenotypes and MEWS score…13
- Supplementary Figure 5: Percentage of explained variances by the dimensions for gamma………………………………………………………………14
- Supplementary Figure 6: Bayesian Information Criterium for the different clustering number and methods for gamma ……………………………………15
- Supplementary Figure 7: Clustering-derived phenotypes for gamma ……......16
-Supplementary Table 2: Clinical, biomarker baseline and outcomes of gamma subclusters…………………………………………………………...……….17

Supplementary Methods

STROBE Statement—checklist of items that should be included in reports of observational studies

	Item No	Recommendation	Page No
**Title and abstract**	1	(*a*) Indicate the study's design with a commonly used term in the title or the abstract	1
		(*b*) Provide in the abstract an informative and balanced summary of what was done and what was found	3
**Introduction**			
Background/rationale	2	Explain the scientific background and rationale for the investigation being reported	4
Objectives	3	State specific objectives, including any prespecified hypotheses	4
**Methods**			
Study design	4	Present key elements of study design early in the paper	5
Setting	5	Describe the setting, locations, and relevant dates, including periods of recruitment, exposure, follow-up, and data collection	5
Participants	6	(*a*) *Cohort study*—Give the eligibility criteria, and the sources and methods of selection of participants. Describe methods of follow-up<br>*Case-control study*—Give the eligibility criteria, and the sources and methods of case ascertainment and control selection. Give the rationale for the choice of cases and controls<br>*Cross-sectional study*—Give the eligibility criteria, and the sources and methods of selection of participants	5
		(*b*) *Cohort study*—For matched studies, give matching criteria and number of exposed and unexposed<br>*Case-control study*—For matched studies, give matching criteria and the number of controls per case	5
Variables	7	Clearly define all outcomes, exposures, predictors, potential confounders, and effect modifiers. Give diagnostic criteria, if applicable	6
Data sources/ measurement	8*	For each variable of interest, give sources of data and details of methods of assessment (measurement). Describe comparability of assessment methods if there is more than one group	*6*
Bias	9	Describe any efforts to address potential sources of bias	6, 11
Study size	10	Explain how the study size was arrived at	7
Quantitative variables	11	Explain how quantitative variables were handled in the analyses. If applicable, describe which groupings were chosen and why	6
Statistical methods	12	(*a*) Describe all statistical methods, including those used to control for confounding	6
		(*b*) Describe any methods used to examine subgroups and interactions	6
		(*c*) Explain how missing data were addressed	6
		(*d*) *Cohort study*—If applicable, explain how loss to follow-up was addressed<br>*Case-control study*—If applicable, explain how matching of cases and controls was addressed<br>*Cross-sectional study*—If applicable, describe analytical methods taking account of sampling strategy	6
		(*e*) Describe any sensitivity analyses	6

Continued on next page

Results

Participants	13*	(a) Report numbers of individuals at each stage of study—eg numbers potentially eligible, examined for eligibility, confirmed eligible, included in the study, completing follow-up, and analysed	8
		(b) Give reasons for non-participation at each stage	8
		(c) Consider use of a flow diagram	8
Descriptive data	14*	(a) Give characteristics of study participants (eg demographic, clinical, social) and information on exposures and potential confounders	8
		(b) Indicate number of participants with missing data for each variable of interest	8
		(c) *Cohort study*—Summarise follow-up time (eg, average and total amount)	8
Outcome data	15*	*Cohort study*—Report numbers of outcome events or summary measures over time	*8*
		Case-control study—Report numbers in each exposure category, or summary measures of exposure	*8*
		Cross-sectional study—Report numbers of outcome events or summary measures	*8*
Main results	16	(*a*) Give unadjusted estimates and, if applicable, confounder-adjusted estimates and their precision (eg, 95% confidence interval). Make clear which confounders were adjusted for and why they were included	8
		(*b*) Report category boundaries when continuous variables were categorized	8
		(*c*) If relevant, consider translating estimates of relative risk into absolute risk for a meaningful time period	8
Other analyses	17	Report other analyses done—eg analyses of subgroups and interactions, and sensitivity analyses	8

Discussion

Key results	18	Summarise key results with reference to study objectives	9,10
Limitations	19	Discuss limitations of the study, taking into account sources of potential bias or imprecision. Discuss both direction and magnitude of any potential bias	11
Interpretation	20	Give a cautious overall interpretation of results considering objectives, limitations, multiplicity of analyses, results from similar studies, and other relevant evidence	9,10
Generalisability	21	Discuss the generalisability (external validity) of the study results	9,10

Other information

Funding	22	Give the source of funding and the role of the funders for the present study and, if applicable, for the original study on which the present article is based	2

*Give information separately for cases and controls in case-control studies and, if applicable, for exposed and unexposed groups in cohort and cross-sectional studies.

Note: An Explanation and Elaboration article discusses each checklist item and gives methodological background and published examples of transparent reporting. The STROBE checklist is best used in conjunction with this article (freely available on the Web sites of PLoS Medicine at http://www.plosmedicine.org/, Annals of Internal Medicine at http://www.annals.org/, and Epidemiology at http://www.epidem.com/). Information on the STROBE Initiative is available at www.strobe-statement.org.

Supplementary Figure 1. Percentage of explained variances by the dimensions resulted from the principal component analysis used for the dimensionality reduction procedure

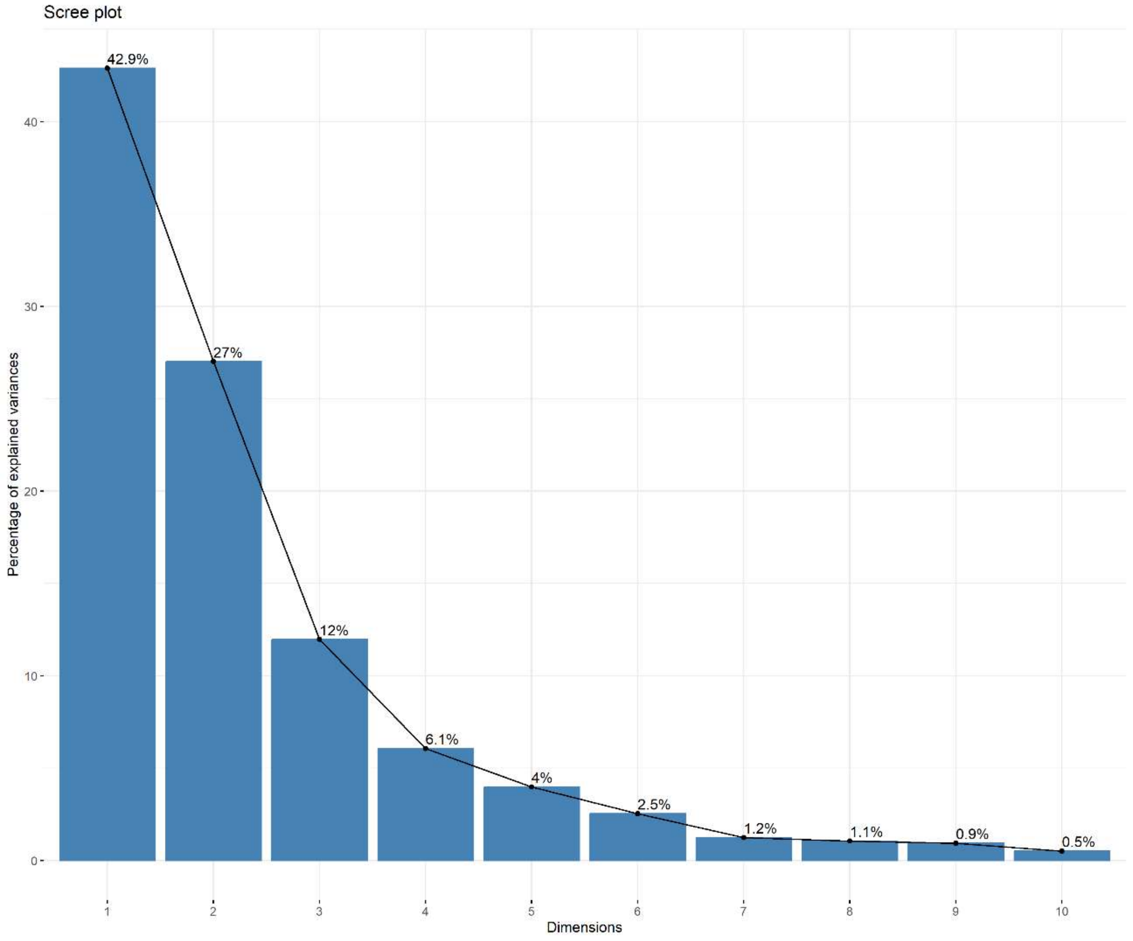

Supplementary Figure 2. Bayesian Information Criterium for the different clustering number and methods. BIC: Bayesian Information criterion; EII: spherical, equal volume; VII: spherical, unequal volume; EEI: diagonal, equal volume and shape; VEI: diagonal, varying volume, equal shape; EVI: diagonal, equal volume, varying shape; VVI: diagonal, varying volume and shape; EEE: ellipsoidal, equal volume, shape, and orientation; VEE: ellipsoidal, equal shape and orientation; EVE: ellipsoidal, equal volume and orientation; VVE: ellipsoidal, equal orientation; EEV: ellipsoidal, equal volume and equal shape; VEV: ellipsoidal, equal shape; EVV: ellipsoidal, equal volume: VVV: ellipsoidal, varying volume, shape, and orientation

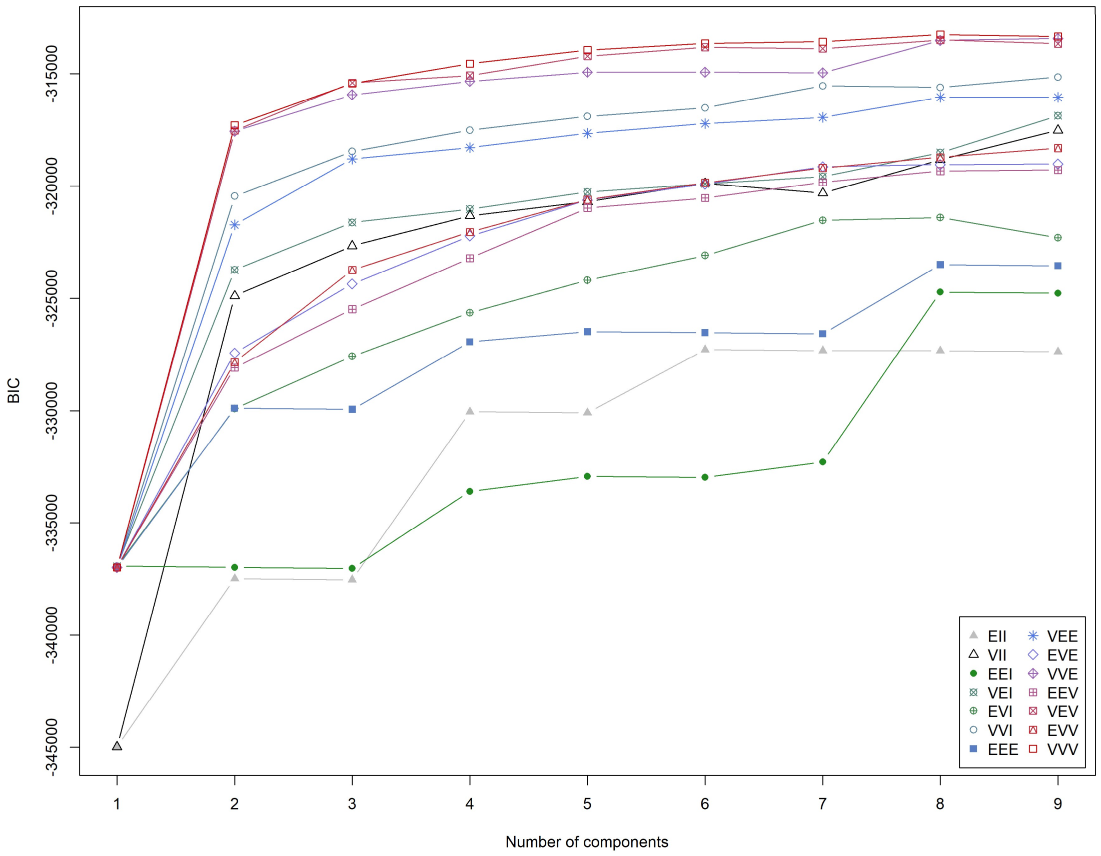

Supplementary Figure 3. Clustering-derived phenotypes. Green=Alpha, Red=Beta, Blue=Gamma phenotype.

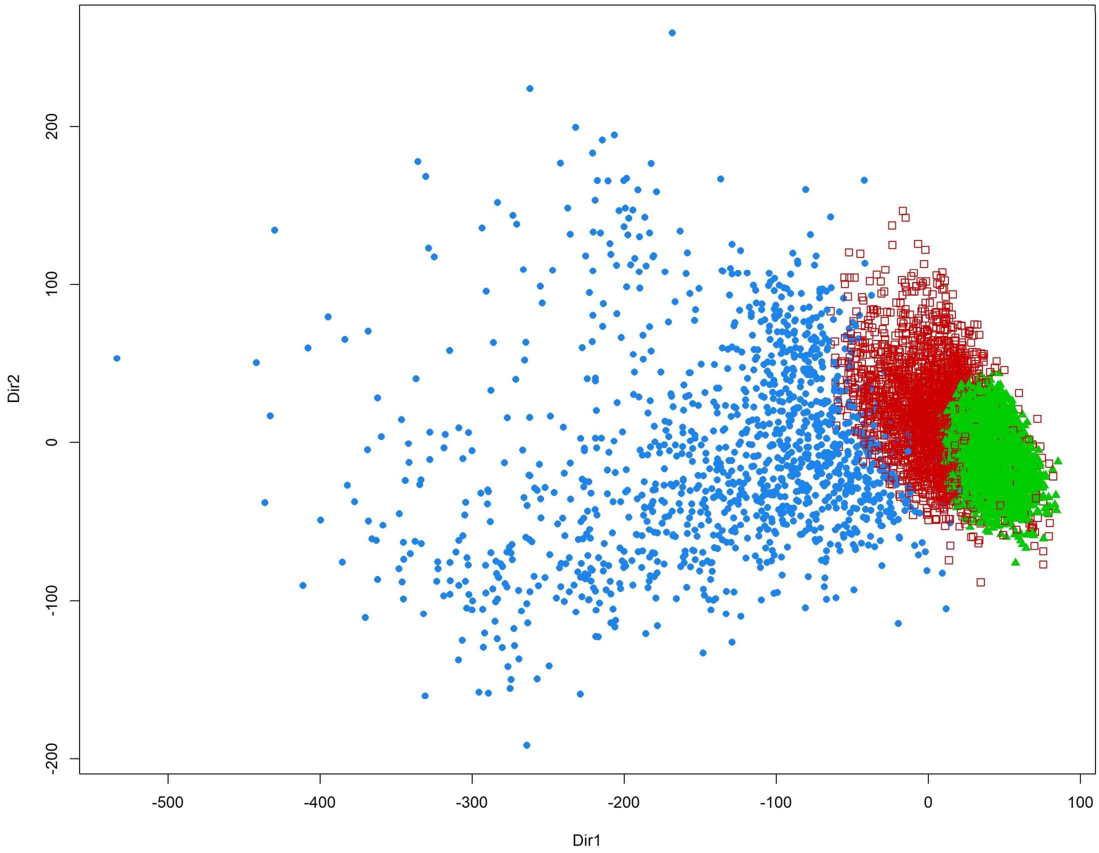

Explanation of clinical criteria for the selection of the number of cluster

According to current literature, early warning scores usually divide risk groups into 3-4 levels (e.g. NEWS2 (1) is divided in four levels of risk, and MEWS (2) in 3 levels of risk). On the other hand, at the clinical level, this classification is very useful and intuitive.

1-Royal College of Physicians. National Early Warning Score (NEWS) 2: Standardising the assessment of acute-illness severity in the NHS. Updated report of a working party. London: RCP 2. 2017.

2-Subbe CP, Davies RG, Williams E, Rutherford P, Gemmell L. Effect of introducing the Modified Early Warning score on clinical outcomes, cardio-pulmonary arrests and intensive care utilization in acute medical admissions. Anaesthesia. 2003 Aug;58(8):797-802. doi: 10.1046/j.1365-2044.2003.03258.x. PMID: 12859475.

Supplementary Table 1: Univariate analysis of mortality

	**Survival**	**Non-survival**	**Hazard Ratio (95% Confidence interval)**	**P value**
	N=7116	***N=793***		
Age, year	63.3 (19.6)	74.8 (15.7)	1.04 [1.03;1.04]	<0.001
Age groups, year:				
18-49	1706 (24.0%)	66 (8.32%)	Ref.	Ref.
50-74	2888 (40.6%)	240 (30.3%)	2.09 [1.59;2.74]	<0.001
>75	2522 (35.4%)	487 (61.4%)	4.59 [3.55;5.93]	<0.001
Transfer:				
No ALS	4430 (62.3%)	633 (79.8%)	Ref.	Ref.
ALS	2686 (37.7%)	160 (20.2%)	0.43 [0.36;0.51]	<0.001
Zone:				
Non-rural	5277 (74.2%)	578 (72.9%)	Ref.	Ref.
Rural	1838 (25.8%)	215 (27.1%)	1.07 [0.92;1.25]	0.387
Nursing homes:				
No	6502 (91.4%)	599 (75.5%)	Ref.	Ref.
Yes	614 (8.63%)	194 (24.5%)	3.11 [2.65;3.66]	<0.001
Respiratory rate, breaths/min	19.2 (7.11)	22.4 (10.7)	1.05 [1.04;1.05]	<0.001
Oxygen saturation, %	95.1 (6.27)	85.4 (14.2)	0.94 [0.93;0.94]	<0.001
Fraction of inspired oxygen, %	0.22 (0.07)	0.28 (0.18)	21.2 [15.0;30.0]	<0.001
SaFi	444 (53.8)	366 (112)	0.99 [0.99;0.99]	<0.001

	Survival	Non-survival	Hazard Ratio (95% Confidence interval)	P value
	N=7116	*N=793*		
Systolic blood pressure, mmHg	136 (30.3)	121 (45.2)	0.98 [0.98;0.99]	<0.001
Diastolic blood pressure, mmHg	78.9 (18.3)	68.0 (27.0)	0.97 [0.97;0.98]	<0.001
Mean blood pressure, mmHg	97.9 (20.7)	85.5 (31.9)	0.98 [0.97;0.98]	<0.001
Heart rate, beats/min	88.3 (27.9)	99.7 (43.0)	1.01 [1.01;1.01]	<0.001
Ocular Glasgow coma scale, points	3.76 (0.66)	2.69 (1.30)	0.42 [0.39;0.44]	<0.001
Verbal Glasgow coma scale, points	4.63 (1.02)	3.12 (1.77)	0.55 [0.53;0.57]	<0.001
Motor Glasgow coma scale, points	5.76 (0.88)	4.23 (2.02)	0.58 [0.56;0.59]	<0.001
Temperature, ºC	36.2 (0.83)	36.2 (1.24)	1.03 [0.95;1.11]	0.534
pH	7.37 (0.10)	7.24 (0.20)	0.01 [0.01;0.01]	<0.001
pCO2, mmHg	41.2 (12.2)	52.8 (22.1)	1.03 [1.03;1.03]	<0.001
pO2, mmHg	33.6 (14.6)	27.3 (15.8)	0.96 [0.96;0.97]	<0.001
Bicarbonate, mEq	24.1 (4.35)	21.4 (6.80)	0.89 [0.88;0.90]	<0.001
Base excess (ecf), mmol/L	-0.31 (4.33)	-4.20 (8.00)	0.89 [0.89;0.90]	<0.001
TCO2, mmol/L	26.1 (5.10)	26.8 (8.44)	1.02 [1.01;1.04]	<0.001
Sodium, mmol/L	139 (3.85)	139 (6.37)	1.01 [0.99;1.03]	0.319
Potassium, mmol/L	4.16 (0.67)	4.58 (1.25)	1.69 [1.60;1.79]	<0.001
Calcium, mmol/L	1.14 (0.11)	1.11 (0.16)	0.10 [0.06;0.17]	<0.001

	Survival	Non-survival	Hazard Ratio (95% Confidence interval)	P value
	N=7116	*N=793*		
Chlorine, mmol/L	103 (4.39)	104 (7.21)	1.04 [1.03;1.05]	<0.001
Hematocrit, %	41.7 (5.95)	39.1 (8.22)	0.94 [0.93;0.95]	<0.001
Hemoglobin, g/dL	14.2 (2.28)	13.2 (2.92)	0.85 [0.83;0.88]	<0.001
Glucose, mg/dL	140 (68.6)	189 (97.8)	1.00 [1.00;1.01]	<0.001
Lactate, mmol/L	2.74 (2.66)	6.26 (4.48)	1.20 [1.18;1.21]	<0.001
Creatinine, mg/dL	1.06 (0.67)	1.87 (1.42)	1.46 [1.42;1.51]	<0.001
Blood urea nitrogen, mg/dL	19.0 (11.8)	34.1 (19.2)	1.02 [1.02;1.03]	<0.001
Osmolarity, mOsm/Kg	292 (9.64)	301 (16.5)	1.04 [1.04;1.05]	<0.001
GAP anion, mmol/L	11.6 (6.03)	13.1 (8.70)	1.04 [1.03;1.05]	<0.001
Urinary anion, mmol/L	39.8 (4.87)	39.0 (7.30)	0.97 [0.96;0.99]	<0.001
Potassium anion, mmol/L	15.7 (6.04)	17.6 (8.88)	1.05 [1.04;1.06]	<0.001
NIMV:				
No	6925 (97.3%)	691 (87.1%)	Ref.	Ref.
Yes	191 (2.68%)	102 (12.9%)	4.53 [3.68;5.58]	<0.001
IMV:				
No	6893 (96.9%)	531 (67.0%)	Ref.	Ref.
Yes	223 (3.13%)	262 (33.0%)	11.3 [9.77;13.1]	<0.001
Vasoactive agents:				

	Survival	Non-survival	Hazard Ratio (95% Confidence interval)	P value
	N=7116	*N=793*		
No	7055 (99.1%)	662 (83.5%)	Ref.	Ref.
Yes	61 (0.86%)	131 (16.5%)	15.5 [12.8;18.7]	<0.001
aCCI, points	4.18 (3.36)	6.96 (3.54)	1.20 [1.18;1.22]	<0.001
Inpatient:				
No	3629 (51.0%)	32 (4.04%)	Ref.	Ref.
Yes	3485 (49.0%)	761 (96.0%)	22.6 [15.8;32.1]	<0.001
ICU-admission:				
No	6526 (91.7%)	487 (61.4%)	Ref.	Ref.
Yes	587 (8.25%)	306 (38.6%)	6.00 [5.20;6.92]	<0.001
ACCU-admission:				
No	6527 (91.7%)	731 (92.2%)	Ref.	Ref.
Yes	587 (8.25%)	62 (7.82%)	0.95 [0.73;1.23]	0.671
Stroke unit-admission:				
No	6734 (94.7%)	755 (95.2%)	Ref.	Ref.
Yes	376 (5.29%)	38 (4.79%)	0.90 [0.65;1.24]	0.508
Phenotype:				
Gamma	4212 (59.2%)	137 (17.3%)	Ref.	Ref.
Beta	2046 (28.8%)	233 (29.4%)	3.37 [2.73;4.16]	<0.001
Alpha	858 (12.1%)	423 (53.3%)	12.8 [10.6;15.6]	<0.001

Abbreviations: NA: not applicable; ALS: advanced life support; SaFi: ratio: pulse oximetry saturation / fraction of inspired oxygen ratio; pCO2: partial pressure of carbon dioxide; pO2: partial pressure of oxygen; TCO2: total carbon dioxide content; NIMV: non-invasive mechanical ventilation; IMV: invasive mechanical ventilation; GB: gastrointestinal bleeding; COPD: chronic obstructive pulmonary disease; SARS-CoV-2: severe acute respiratory syndrome coronavirus 2; aCCI: Age adjusted Charlson comorbidity index; ICU: intensive care unit; ACCU: Acute Cardiac Care Unit.
Values expressed as total number (percentage) and medians (25 percentile-75 percentile), as appropriate.

Supplementary Figure 4: Survival curves of phenotypes and MEWS score. Blue line = low-risk MEWS, Red line = gamma phenotype, dark blue = intermediate-risk MEWS, yellow line = beta phenotype; pink line = high-risk MEWS, green line = alpha phenotype.

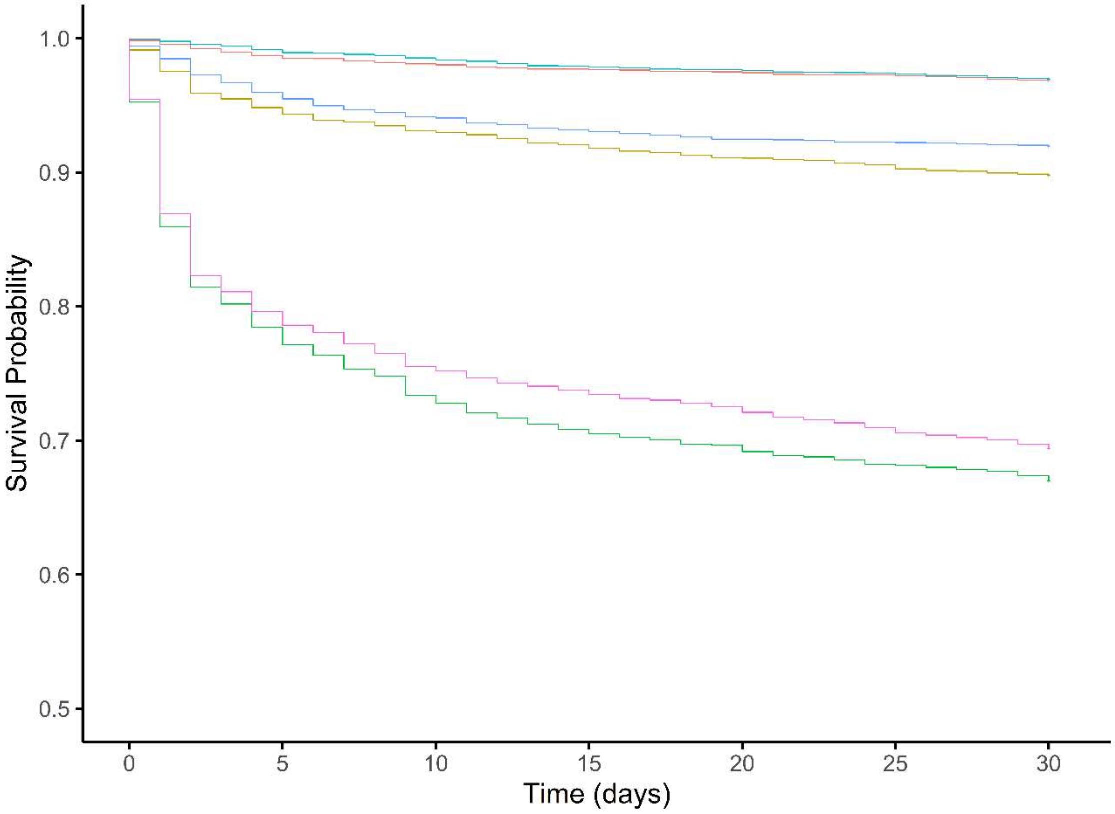

Supplementary Figure 5. Percentage of explained variances by the dimensions resulted from the principal component analysis used for the dimensionality reduction procedure

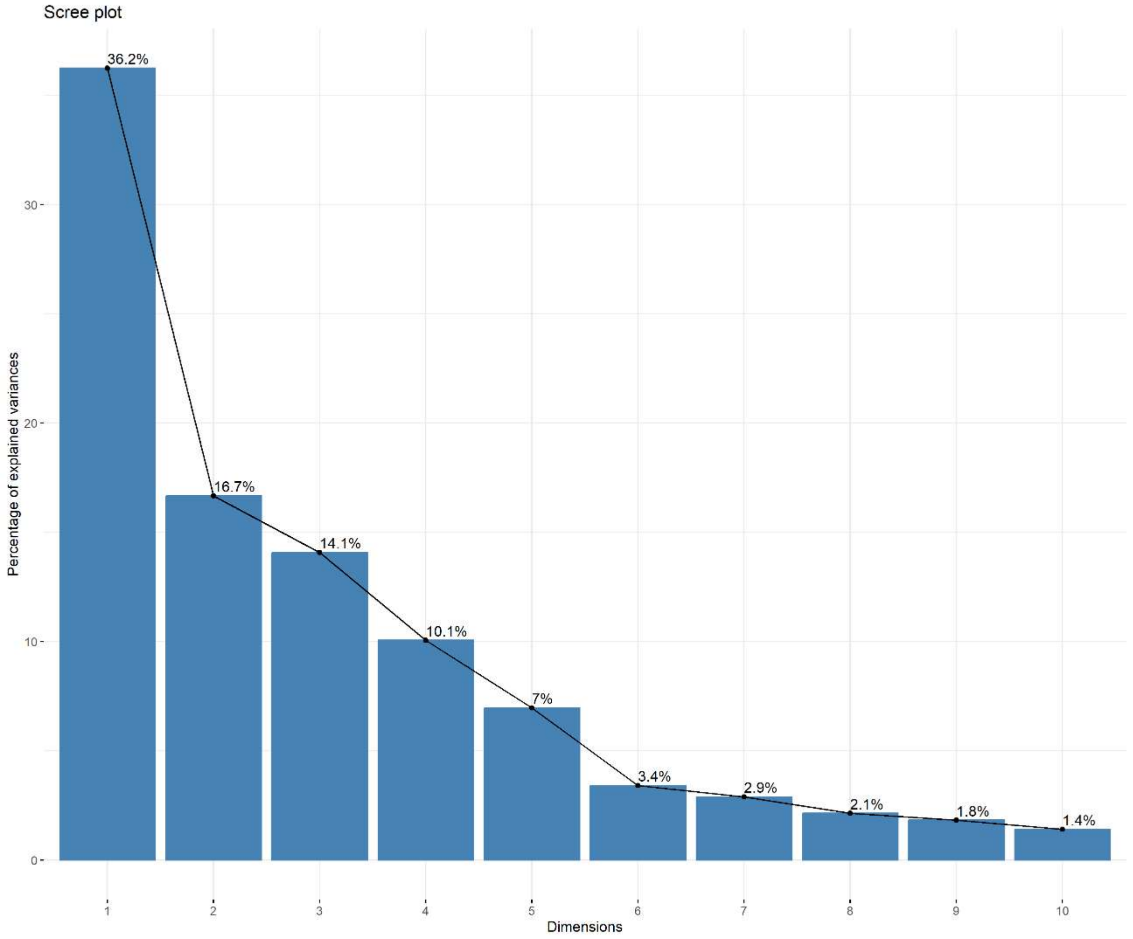

Supplementary Figure 6. Bayesian Information Criterium for the different clustering number and methods. BIC: Bayesian Information criterion; EII: spherical, equal volume; VII: spherical, unequal volume; EEI: diagonal, equal volume and shape; VEI: diagonal, varying volume, equal shape; EVI: diagonal, equal volume, varying shape; VVI: diagonal, varying volume and shape; EEE: ellipsoidal, equal volume, shape, and orientation; VEE: ellipsoidal, equal shape and orientation; EVE: ellipsoidal, equal volume and orientation; VVE: ellipsoidal, equal orientation; EEV: ellipsoidal, equal volume and equal shape; VEV: ellipsoidal, equal shape; EVV: ellipsoidal, equal volume: VVV: ellipsoidal, varying volume, shape, and orientation

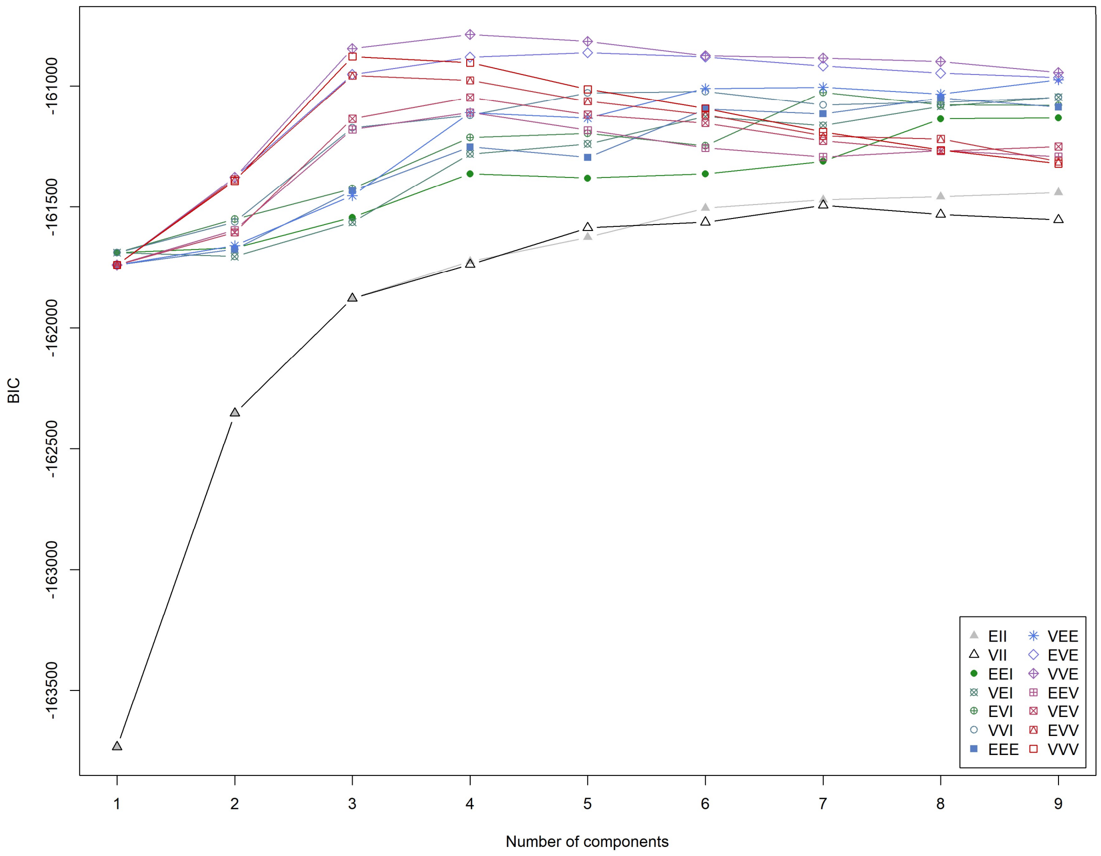

Supplementary Figure 7. Clustering-derived phenotypes. Green=Alpha, Red=Beta, Blue=Gamma phenotype.

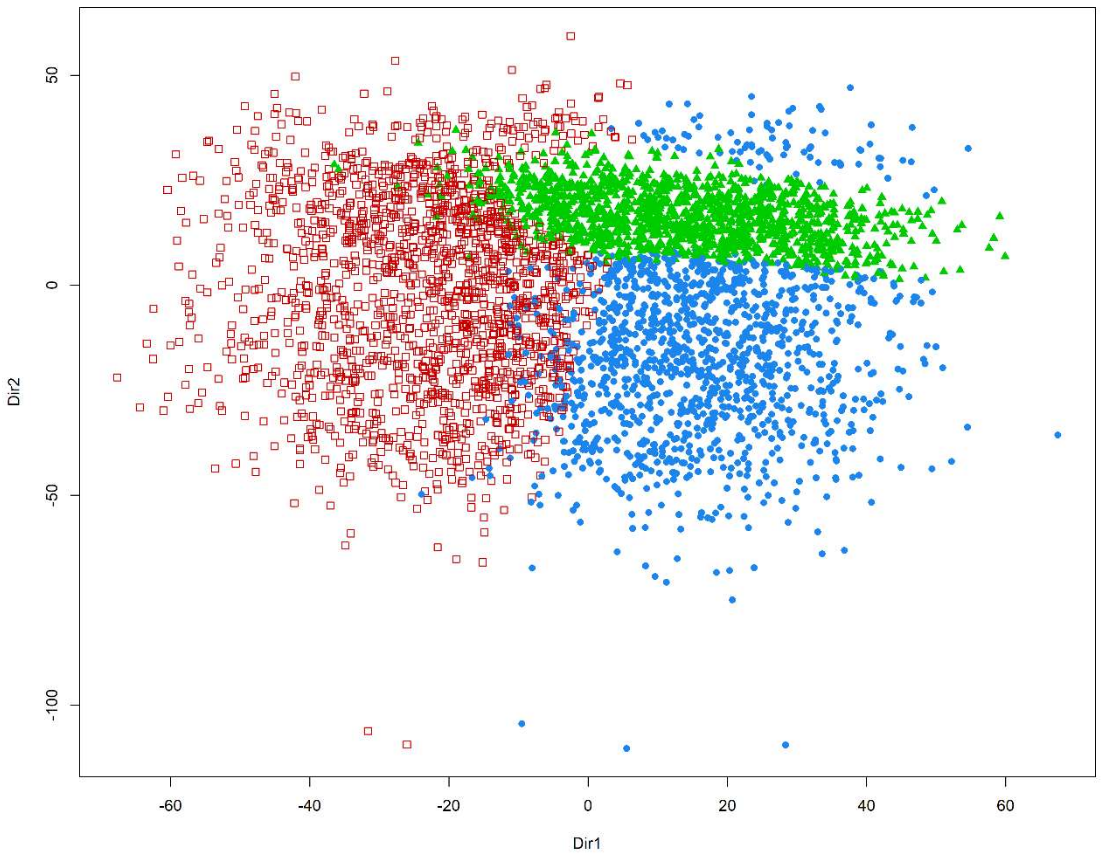

Supplementary Table 2: Clinical, biomarker baseline and outcomes of gamma subclusters

	Gamma #1 *N=1321*	Gamma #2 *N=1704*	Gamma #3 *N=1324*	p.value
Age, year	74.1 (11.6)	42.6 (14.5)	71.1 (13.8)	0.000
Age groups, year:				0.000
18-49	30 (2.27%)	1141 (67.0%)	82 (6.19%)	
50-74	589 (44.6%)	536 (31.5%)	615 (46.5%)	
>75	702 (53.1%)	27 (1.58%)	627 (47.4%)	
Sex:				0.472
Male	777 (58.8%)	965 (56.6%)	767 (57.9%)	
Female	544 (41.2%)	739 (43.4%)	557 (42.1%)	
Transfer:				0.598
No ALS	787 (59.6%)	984 (57.7%)	774 (58.5%)	
ALS	534 (40.4%)	720 (42.3%)	550 (41.5%)	
Zone:				<0.001
Non-rural	1026 (77.7%)	1211 (71.1%)	1002 (75.7%)	
Rural	295 (22.3%)	492 (28.9%)	322 (24.3%)	
Nursing homes:				<0.001
No	1187 (89.9%)	1678 (98.5%)	1223 (92.4%)	
Yes	134 (10.1%)	26 (1.53%)	101 (7.63%)	
Respiratory rate, breaths/min	17.6 (5.54)	18.4 (5.69)	16.9 (4.43)	<0.001
Oxygen saturation, %	96.8 (1.89)	97.9 (1.72)	97.0 (1.77)	<0.001
Fraction of inspired oxygen, %	0.21 (0.00)	0.21 (0.00)	0.21 (0.00)	0.318
SaFi	461 (9.01)	466 (8.18)	462 (8.43)	<0.001
Systolic blood pressure, mmHg	145 (33.8)	129 (18.5)	141 (30.6)	<0.001
Diastolic blood pressure, mmHg	80.0 (19.2)	80.2 (14.0)	79.3 (18.2)	0.342
Mean blood pressure, mmHg	102 (22.5)	96.4 (14.1)	99.8 (20.7)	<0.001
Heart rate, beats/min	71.8 (15.7)	88.6 (16.6)	74.7 (16.8)	<0.001
Ocular Glasgow coma scale, points	3.84 (0.54)	3.81 (0.58)	3.84 (0.53)	0.188
Verbal Glasgow coma scale, points	4.73 (0.89)	4.71 (0.89)	4.71 (0.91)	0.868

	Gamma #1 *N=1321*	Gamma #2 *N=1704*	Gamma #3 *N=1324*	p.value
Motor Glasgow coma scale, points	5.85 (0.68)	5.82 (0.76)	5.83 (0.73)	0.571
Temperature, ºC	36.1 (0.74)	36.2 (0.69)	36.1 (0.67)	0.002
pH	7.40 (0.07)	7.39 (0.08)	7.37 (0.07)	<0.001
pCO2, mmHg	38.2 (8.94)	37.6 (9.27)	42.0 (7.83)	<0.001
pO2, mmHg	41.3 (14.5)	36.3 (14.3)	24.6 (4.31)	<0.001
Bicarbonate, mEq	24.5 (3.20)	23.8 (3.79)	24.5 (3.82)	<0.001
Base excess (ecf), mmol/L	0.25 (2.97)	-0.39 (3.75)	0.30 (3.31)	<0.001
TCO2, mmol/L	25.1 (3.96)	24.8 (4.29)	27.9 (4.19)	<0.001
Sodium, mmol/L	139 (3.66)	139 (3.31)	139 (3.44)	0.007
Potassium, mmol/L	4.13 (0.60)	4.09 (0.61)	4.15 (0.63)	0.030
Calcium, mmol/L	1.15 (0.11)	1.14 (0.10)	1.13 (0.10)	<0.001
Chlorine, mmol/L	104 (4.73)	103 (3.76)	103 (3.75)	<0.001
Hematocrit, %	41.5 (5.65)	42.2 (5.22)	41.5 (5.36)	<0.001
Hemoglobin, g/dL	14.1 (2.17)	14.4 (2.07)	14.1 (2.12)	<0.001
Glucose, mg/dL	122 (21.7)	107 (19.9)	118 (23.2)	<0.001
Lactate, mmol/L	2.06 (1.58)	2.71 (2.87)	2.32 (2.22)	<0.001
Creatinine, mg/dL	1.07 (0.64)	0.86 (0.42)	1.02 (0.60)	<0.001
Blood urea nitrogen, mg/dL	19.3 (10.8)	14.3 (7.02)	18.5 (10.6)	<0.001
Osmolarity, mOsm/Kg	292 (8.52)	289 (7.00)	291 (7.91)	<0.001
GAP anion, mmol/L	11.0 (5.51)	12.1 (5.36)	11.5 (5.27)	<0.001
Urinary anion, mmol/L	39.6 (5.06)	40.0 (4.33)	40.2 (4.48)	0.006
Potassium anion, mmol/L	15.1 (5.55)	16.2 (5.40)	15.6 (5.30)	<0.001
NIMV:				0.021
No	1316 (99.6%)	1703 (99.9%)	1324 (100%)	
Yes	5 (0.38%)	1 (0.06%)	0 (0.00%)	
IMV:				0.731
No	1295 (98.0%)	1667 (97.8%)	1292 (97.6%)	
Yes	26 (1.97%)	37 (2.17%)	32 (2.42%)	

	Gamma #1 *N=1321*	Gamma #2 *N=1704*	Gamma #3 *N=1324*	p.value
Vasoactive agents:				0.174
No	1318 (99.8%)	1702 (99.9%)	1318 (99.5%)	
Yes	3 (0.23%)	2 (0.12%)	6 (0.45%)	
aCCI, points	5.14 (2.78)	1.36 (2.12)	4.69 (2.93)	0.000
Inpatient:				<0.001
No	664 (50.3%)	1174 (69.0%)	716 (54.1%)	
Yes	657 (49.7%)	528 (31.0%)	608 (45.9%)	
ICU-admission:				0.989
No	1237 (93.6%)	1596 (93.8%)	1241 (93.7%)	
Yes	84 (6.36%)	106 (6.23%)	83 (6.27%)	
ACCU-admission:				<0.001
No	1184 (89.6%)	1641 (96.4%)	1197 (90.4%)	
Yes	137 (10.4%)	61 (3.58%)	127 (9.59%)	
Días.ingreso	4.86 (9.47)	2.94 (6.86)	4.37 (8.98)	<0.001
2-day mortality:				0.006
No	1306 (98.9%)	1700 (99.8%)	1310 (98.9%)	
Yes	15 (1.14%)	4 (0.23%)	14 (1.06%)	
7-day mortality:				<0.001
No	1284 (97.2%)	1696 (99.5%)	1295 (97.8%)	
Yes	37 (2.80%)	8 (0.47%)	29 (2.19%)	
30-day mortality:				<0.001
No	1250 (94.6%)	1690 (99.2%)	1272 (96.1%)	
Yes	71 (5.37%)	14 (0.82%)	52 (3.93%)	

Abbreviations: NA: not applicable; ALS: advanced life support; SaFi: ratio: pulse oximetry saturation / fraction of inspired oxygen ratio; pCO2: partial pressure of carbon dioxide; pO2: partial pressure of oxygen; TCO2: total carbon dioxide content; NIMV: non-invasive mechanical ventilation; IMV: invasive mechanical ventilation; GB: gastrointestinal bleeding; COPD: chronic obstructive pulmonary disease; SARS-CoV-2: severe acute respiratory syndrome coronavirus 2; aCCI: Age adjusted Charlson comorbidity index; ICU: intensive care unit; ACCU: Acute Cardiac Care Unit.
Values expressed as total number (percentage) and medians (25 percentile-75 percentile), as appropriate.
