# Supplementary material for: Clinical phenotypes and short-term outcomes based on prehospital point-of-care testing and on-scene vital signs
Source: NPJ Digit Med. 2024 Jul 24;7:197. doi: 10.1038/s41746-024-01194-6 (PMC11269726; doi:10.1038/s41746-024-01194-6)
Supplement: Supplementary file 2 — Reporting Summary [file 41746_2024_1194_MOESM2_ESM.pdf]

nature portfolio

Corresponding author(s): Ancor Sanz-Garcia

Last updated by author(s): Apr 24, 2024

Reporting Summary

Nature Portfolio wishes to improve the reproducibility of the work that we publish. This form provides structure for consistency and transparency in reporting. For further information on Nature Portfolio policies, see our Editorial Policies and the Editorial Policy Checklist.

Statistics

For all statistical analyses, confirm that the following items are present in the figure legend, table legend, main text, or Methods section.

n/a	Confirmed	
☐	☒	The exact sample size (*n*) for each experimental group/condition, given as a discrete number and unit of measurement
☐	☒	A statement on whether measurements were taken from distinct samples or whether the same sample was measured repeatedly
☐	☒	The statistical test(s) used AND whether they are one- or two-sided<br>*Only common tests should be described solely by name; describe more complex techniques in the Methods section.*
☐	☒	A description of all covariates tested
☐	☒	A description of any assumptions or corrections, such as tests of normality and adjustment for multiple comparisons
☐	☒	A full description of the statistical parameters including central tendency (e.g. means) or other basic estimates (e.g. regression coefficient) AND variation (e.g. standard deviation) or associated estimates of uncertainty (e.g. confidence intervals)
☐	☒	For null hypothesis testing, the test statistic (e.g. *F*, *t*, *r*) with confidence intervals, effect sizes, degrees of freedom and *P* value noted<br>*Give P values as exact values whenever suitable.*
☒	☐	For Bayesian analysis, information on the choice of priors and Markov chain Monte Carlo settings
☐	☒	For hierarchical and complex designs, identification of the appropriate level for tests and full reporting of outcomes
☒	☐	Estimates of effect sizes (e.g. Cohen's *d*, Pearson's *r*), indicating how they were calculated

Our web collection on statistics for biologists contains articles on many of the points above.

Software and code

Policy information about availability of computer code

Data collection	IBM SPSS Statistics for Apple version 20.0 software. (IBM Corp, Armonk, NY, USA)
Data analysis	All calculations and analyses were performed by our own codes, R packages

For manuscripts utilizing custom algorithms or software that are central to the research but not yet described in published literature, software must be made available to editors and reviewers. We strongly encourage code deposition in a community repository (e.g. GitHub). See the Nature Portfolio guidelines for submitting code & software for further information.

Data

Policy information about availability of data

All manuscripts must include a data availability statement. This statement should provide the following information, where applicable:

- Accession codes, unique identifiers, or web links for publicly available datasets
- A description of any restrictions on data availability
- For clinical datasets or third party data, please ensure that the statement adheres to our policy

The datasets used and/or analyzed during the current study are available from the corresponding author upon reasonable request

Research involving human participants, their data, or biological material

Policy information about studies with human participants or human data. See also policy information about sex, gender (identity/presentation), and sexual orientation and race, ethnicity and racism.

Reporting on sex and gender	Sex has been provided and included in the analyses
Reporting on race, ethnicity, or other socially relevant groupings	Race, ethnicity or other relevant groupings were not collected
Population characteristics	The median (IQR) age was 64 (51-80) years, 41% were women, and 26% were living in rural areas (Table 1). Clinical characterization via unsupervised machine learning revealed three clinical phenotypes that exhibited marked differences.
Recruitment	From an original population of 11,182 patients, 8,136 were considered eligible, and 7,909 (97·2%) subjects satisfied the inclusion criteria and were included in the final cohort analysis
Ethics oversight	Patients were prospectively included uninterruptedly from two studies conducted under identical research protocols, the "HITS study" (ISRCTN48326533) and the preBIO study" (ISRCTN49321933), which were approved by the institutional review board of the Public Health Service and followed the STrengthening the Reporting of OBservational studies in Epidemiology (STROBE) statement (supplementary material p3)

Note that full information on the approval of the study protocol must also be provided in the manuscript.

Field-specific reporting

Please select the one below that is the best fit for your research. If you are not sure, read the appropriate sections before making your selection.

☒ Life sciences ☐ Behavioural & social sciences ☐ Ecological, evolutionary & environmental sciences

For a reference copy of the document with all sections, see nature.com/documents/nr-reporting-summary-flat.pdf

Life sciences study design

All studies must disclose on these points even when the disclosure is negative.

Sample size	The sample size needed for the clustering studies has been recently estimated (29). Due to the characteristics of the clustering procedure, the phenotypes derived from clustering are driven by large effect sizes or by the accumulation of small effect sizes among the multiple variables analyzed, and there is no effect of the covariance structure difference. Therefore, a small sample size (e.g., N=20), as stated in (29), allows large cluster separations.
Data exclusions	Missing values were random; therefore, a listwise deletion method was used since it does not induce biased means, variances or regression weight modifications
Replication	The results were compared to already developed scores
Randomization	This is not relevant for the study since the unsupervised machine learning method is blind to the outcome
Blinding	This is not relevant for the study since the unsupervised machine learning method is blind to the outcome

Reporting for specific materials, systems and methods

We require information from authors about some types of materials, experimental systems and methods used in many studies. Here, indicate whether each material, system or method listed is relevant to your study. If you are not sure if a list item applies to your research, read the appropriate section before selecting a response.

Materials & experimental systems

n/a	Involved in the study	
☒	☐	Antibodies
☒	☐	Eukaryotic cell lines
☒	☐	Palaeontology and archaeology
☒	☐	Animals and other organisms
☐	☒	Clinical data
☒	☐	Dual use research of concern
☒	☐	Plants

Methods

n/a	Involved in the study	
☒	☐	ChIP-seq
☒	☐	Flow cytometry
☒	☐	MRI-based neuroimaging

Clinical data

Policy information about clinical studies

All manuscripts should comply with the ICMJE guidelines for publication of clinical research and a completed CONSORT checklist must be included with all submissions.

Clinical trial registration	Patients were prospectively included uninterruptedly from two studies conducted under identical research protocols, the "HITS study" (ISRCTN48326533) and the preBIO study" (ISRCTN49321933)
Study protocol	(ISRCTN48326533) and (ISRCTN49321933)
Data collection	Covariates included sociodemographic variables (sex at birth and age); on-scene vital signs (respiratory rate, oxygen saturation, blood pressure, heart rate, temperature, and Glasgow coma scale); and prehospital blood analysis (pH, bicarbonate, excess bases, sodium, potassium, chloride, calcium, hemoglobin, hematocrit, creatinine, blood urea nitrogen, glucose, and lactate), which were obtained by the ERN based on the first encounter. Vital signs were obtained via a LifePAK® 15 monitor-defibrillator (Physio-Control, Inc., Redmond, USA), and blood tests were performed by means of an Epoc® analyzer (Siemens Healthcare GmbH, Erlangen, Germany). The respiratory rate was monitored by direct observation and counting of breathing cycles for half a minute; in the case of very shallow or difficult breathing, the respiratory rate was measured by direct auscultation. Oxygen therapy (by any method) was also administered at the time of the patient's diagnosis of ALS; once the fraction of inspired oxygen was known, the pulse oximetry saturation/fraction of inspired oxygen ratio (SaFi) was calculated.<br>After a 30-day follow-up period, data on mortality, comorbidities and hospital admissions were collected by reviewing the patients' electronic medical records. The data were recorded electronically in a database specifically designed for this purpose, recording the prehospital care variables. Access was provided by individual passwords and double authentication. After the data were cleaned (logic, range and consistency tests), a total of 54 variables were analyzed. Once the data were linked, patient identifiers were anonymized.
Outcomes	The principal outcome was cumulative mortality (all-cause) at 2, 7, and 30 days. The secondary variables considered included on-scene life support interventions (advanced airway management, defibrillation or pacemaker application, and intravenous medication delivery), suspected prehospital diagnoses (29 different subcategories), hospital outcomes (inpatient, intensive care unit admission), and 17 comorbidities needed to calculate the age-adjusted Charlson comorbidity index (aCCI).

Plants

Seed stocks	NA
Novel plant genotypes	NA
Authentication	NA
